# Causal Relationship between Adiponectin and Diabetic Retinopathy: A Mendelian Randomization Study in an Asian Population

**DOI:** 10.3390/genes12010017

**Published:** 2020-12-24

**Authors:** Yu-Chuen Huang, Ya-Wen Chang, Chun-Wen Cheng, Chia-Ming Wu, Wen-Ling Liao, Fuu-Jen Tsai

**Affiliations:** 1 School of Chinese Medicine, China Medical University, Taichung 404, Taiwan; yuchuen@mail.cmu.edu.tw; 2 Genetic Center, Department of Medical Research, China Medical University Hospital, China Medical University, Taichung 404, Taiwan; windy87518@gmail.com (Y.-W.C.); chiaming119@gmail.com (C.-M.W.); 3 Institute of Medicine, Chung Shan Medical University, Taichung 404, Taiwan; cwcheng@csmu.edu.tw; 4 Clinical Laboratory, Chung Shan Medical University Hospital, Taichung 404, Taiwan; 5 Graduate Institute of Integrated Medicine, China Medical University, Taichung 404, Taiwan; 6 Center for Personalized Medicine, China Medical University Hospital, Taichung 404, Taiwan; 7 Division of Pediatric Genetics, Children’s Hospital of China Medical University, Taichung 404, Taiwan

**Keywords:** adiponectin, diabetic retinopathy, Mendelian randomization, causal relationship

## Abstract

Adiponectin (APN) is suggested to be a potential biomarker for predicting diabetic retinopathy (DR) risk, but the association between APN and DR has been inconsistent in observational studies. We used a Mendelian randomization (MR) analysis to evaluate if circulating APN levels result in DR. We applied three different genetic risk scores (GRS): GRS_All_ combined all 47 single nucleotide polymorphisms (SNPs), which from a genome-wide association study (GWAS) database-catalog reach significance level; GRS_Limited_ comprised 16 GRS_All_-SNPs with a rigorous threshold (*p* < 5.0 × 10^−8^ for GWAS), and GRS_APN_ combined 5 SNPs significantly associated with APN level. The MR-inverse-variance weighted method analysis showed that for each 1-SD increase in genetically induced increase in plasma APN, the OR of having DR was β = 0.20 (95% CI: −0.46–0.85, *p* = 0.553) for GRS_APN_, 0.61 (95% CI: 0.10–1.13, *p* = 0.020) for GRS_All_, and 0.57 (95% CI: −0.06 to 1.20, *p* = 0.078) for GRS_Limited_. Sensitivity analysis, including MR-egger regression and the weighted-median approach, did not provide evidence of the pleiotropic effect of IVs. Limited evidence for the causal role of APN in DR risk among Taiwanese diabetic patients was shown based on MR analysis in the present study.

## 1. Introduction

Diabetic retinopathy (DR) is a major microvascular complication in patients with type II diabetes (T2D). The prevalence of DR is approximately 22% among T2D patients and up to 80% of patients with 10 or more years of diabetes have DR. Given its high prevalence, DR is a significant public health problem. It is responsible for a large proportion of blindness in the population, and negatively impacts both the overall healthcare expenditure and patients’ quality of life [1,2]. After the initial diagnosis of T2D, an eye examination with dilatation by an ophthalmologist or optometrist, with subsequent annual visits is recommended for the early detection of DR. Research shows that early detection of non-proliferative DR (NPDR) may lead to a 60% reduction in proliferative DR (PDR), and a 83% reduction in blindness [3]. However, mild NPDR usually does not affect vision, and goes unnoticed and undiagnosed in many diabetic patients.

Adiponectin (APN) is a cytokine secreted by fat cells or adipocytes into the bloodstream. It is involved in different pathways that regulate insulin sensitivity [4,5], inflammation [6], and atherogenesis [7]. Previous studies have shown that a higher APN level is associated with a lower risk of DM development, and a cardio-protective effect [8,9]. Also, evidence suggests that APN supports vascular maintenance, indicating a potentially protective role against DR in T2D patients [10,11]. Therefore, increasing APN levels through medicinal supplements may be beneficial for T2D subjects to prevent the development of DR [12,13,14,15]. Yet, observational studies report inconsistent associations between APN and DR (8–10). Both positive and inverse associations between APN and DR progression, with small standard mean difference values (+0.38 to −0.62), have been reported by meta-analysis studies which combined various ethnic groups [16,17]. Therefore, it is currently unclear whether an elevated APN is a beneficial response, or a compensatory mechanism to counter inflammatory processes that occur as a part of the pathophysiology of microvascular disease complication [16]. We think that contradictory results could arise as a result of confounding, measurement biases, or reverse causation. Previous studies have shown that APN could be influenced by factors such as age, gender, obesity, blood lipid and glucose levels, renal function, smoking, the form of APN measured, and anti-diabetic/cardiovascular drugs. Also, the APN protein is encoded by the *ADIPOQ* gene located on chromosome 3q27 and the association between *ADIPOQ* genetic polymorphisms and serum APN level was observed [18,19].

Hence, it is crucial to distinguish if APN has a causal role in DR, or if it is a marker of the underlying mechanisms. Resolution of this question will improve our understanding of DR. Mendelian randomization (MR) has been used to investigate possible causal relationships of an intermediate trait related to a disease [16,20]. MR is unaffected by confoundment from environmental factors, because it takes advantage of the random allocation of alleles at conception [21], and can substantially improve causal inference in observational data. In the current study, we performed a MR to assess the causal relationship between APN and DR, by using the single-nucleotide polymorphisms (SNPs) most commonly associated with APN as instrumental variables (IVs).

## 2. Materials and Methods

### 2.1. Study Design

We performed an MR analysis to test the causal effect of APN on DR risk. Data on the associations of SNPs with APN levels and DR risk were combined to estimate the influence of blood APN on DR risk. We also analyzed the association of selected APN-related SNPs with a range of DR risk factors; including gender, systolic blood pressure (SBP), diastolic blood pressure (DBP), glucose, HbA1c, triglyceride (TG), high-density lipoprotein cholesterol (HDL), low-density lipoprotein cholesterol (LDL), and body mass index (BMI) to determine the presence of potential bias (horizontal pleiotropy) or mediation of the effect of APN on DR via other DR risk factors (vertical pleiotropy).

### 2.2. Data Source

T2D patients aged over 20 years (*n* = 1251) were recruited from the Taiwan Biobank (TWB) (http://www.twbiobank.org.tw/new_web/about-export.php) and China Medical University Hospital (CMUH), Taichung, Taiwan. TWB is the first and largest government-supported biobank in Taiwan [22]. A total of 29 TWB recruitment centers are located in each city or county of Taiwan. Since 2012, TWB has continuously recruited community-based volunteers who are of 30 to 70 years of age and have no history of cancers. All participants signed an informed consent form, and provided blood samples and a range of personal/lifestyle information via a face-to-face interview and physical examination [22,23,24] at the enrollment and follow-up. Subjects with any one of following conditions were defined as T2D subjects: (1) self-reported T2D, (2) fasting glucose ≥ 126 mg/dL, or (3) HbA1c ≥ 6.5%. DR status was self-reported (by asking “have you been identified with retinopathy?”) and confirmed by expert ophthalmologists for part of subjects recruited from hospital according to the International Clinical DR Disease Severity Scale proposed by the American Academy of Ophthalmology [25]. Detailed information about the number of subjects from each source, and the subject numbers confirmed by ophthalmologists is provided in Table S1. The relevant demographics and plasma samples of the participants were obtained from the TWB, or collected during enrollment at CMUH. The genome wide genotyping was done using the HumanHap550-Duo BeadChip (Illumina, San Diego, CA, USA) or TWBv.1 chip (Thermo Fisher Scientific, Waltham, MA, USA).

The study protocol was approved by the Institutional Review Board of CMUH (CMUH103-REC2-071, CR-3) and Academia Sinica for the Protection of Human, the date of approval for the original version was 2 August 2014. Informed consent was obtained from all participants. We confirm that all experiments of the study were performed in accordance with relevant guidelines and regulations.

### 2.3. Measurements

#### 2.3.1. Anthropometric and Laboratory Measurements

Anthropometric measurements (weight, height, and waist circumference), SBP/DBP, laboratory values (blood lipid, glucose, HbA1c, creatinine, urine albumin), smoking status, and the use of anti-diabetic and cardiovascular drugs were obtained from a self-reported questionnaire.

#### 2.3.2. Plasma APN Level Measurements

The plasma APN level was measured by an enzyme-linked immunosorbent assay (ELISA) kit (Invitrogen, Waltham, MA, USA). The sensitivity of the assay is 100 pg/mL, with the intra-assay and inter-assay coefficient of variation being 3.5 and 5.8%, respectively. Plasma samples or standards were added to each microtiter plate well with a biotin-conjugated antibody specific to the protein being identified. The assay was performed according to the manufacturers’ instructions and optical density (OD) was measured at 405 nm. The concentration of protein in the samples was calculated by fitting the OD values into a standard curve.

### 2.4. Genetic Instrument Variables (IV)

A total of 68 SNPs that were associated (*p* < 0.05) with blood APN levels in subjects with East Asian ancestry (China, Hong Kong, Japan, Korea, and Taiwan) were identified from the genome-wide association studies (GWAS) consortia, or by hand searching existing literature (Table S2). Compiled summary statistics were either downloaded from the NHGRI-EBI GWAS catalog (Buniello, MacArthur et al., 2019) on 1 November 2018, or extracted from each individual study. A total of 47 independent SNPs were selected by linkage disequilibrium pruning of the 68 SNPs, retaining SNPs that explained the most variance in APN levels in each linkage disequilibrium block (linkage disequilibrium threshold; *R*2 < 0.05 in HapMap CHB population) (Table S3).

We replicated the 47 selected SNPs in our study population. For selected SNPs that were not on the HumanHap550-Duo BeadChip or TWB chip, genotype imputation was performed according to the methodology of Howie et al. [26] in IMPUTE version 2 (http://mathgen.stats.ox.ac.uk/impute/impute_v2.html). The panel from the 1000 Genomes Project was used as the reference for imputation to choose the best customized reference set for each individual. SNPs with low imputation quality (INFO < 0.4) or Minor allele frequency (MAF) < 0.5% were excluded from further analysis.

Multiple independent SNPs were combined into three genetic risk scores (GRS) as instrumental variables, including the: Aforementioned 47 independent SNPs (GRS_All_); 5 independent SNPs significantly associated with APN levels (GRS_APN_); and 16 independent SNPs which comprised GRS_All_-SNPs limited with a rigorous threshold (*p* < 5.0 × 10^−8^ for GWAS and *p* < 0.05 for case-control or meta-analysis studies) (GRS_Limited_). The detailed information pertaining to the selection of SNPs for each IV are shown in Table S4 [27]. The details of GRS calculation are described elsewhere [28,29]. To ensure the strength of genetic methodology, we calculated the F-statistics for each individual genetic instrument, with a F-value above 10 indicating that a causal estimate was unlikely to be biased due to weak instruments. A flow diagram is shown in Figure S1 which describes the process for identifying genetic variants.

### 2.5. Statistical Analysis

Continuous data are presented as means with standard deviation, and categorical data are presented as proportions. We used T-tests to compare the mean values of continuous variables, and chi-squared tests to compare the frequencies of categorical variables between two groups. APN values were log transformed to achieve approximate normal distribution. An inverse variance weighted average of associations for specific polymorphisms was used for MR study. SNP association with APN level was tested using multiple linear regression analyses using an additive genetic model. The association between plasma APN and DR at each SNP was calculated as β(APN-DR) = β(SNP-APN)/β(SNP-DR), where β(APN-DR) represents the estimated effect size (logarithm of the odds ratio [OR]) of 1 SD of genetically determined plasma APN levels on DR. The combined β (APN-DR) values from all SNPs were estimated by inverse-variance weighted (IVW) meta-analysis. The sensitivity analyses, including MR-Egger regression and weighted-median approach were performed. All statistical analyses were performed using SPSS v. 22.0 for Windows (IBM, Armonk, NY, USA) or R version 3.4.4 (R Core Team, Vienna, Austria, 2018).

## 3. Results

### 3.1. Characteristics of the Study Participants

Among the 1251 T2D patients included in this study, 438 had DR (cases) and 813 did not (controls). We observed no significant differences in the gender distribution or in the mean value of TG and HDL between the two groups. Age, blood pressure (systolic and diastolic), HbA1c, glucose, and APN in cases were significantly higher than that of the controls (*p* < 0.01, Table 1). The DR cases also had lower BMI and LDL compared to the controls (*p* = 0.01 and <0.001, respectively, Table 1).

### 3.2. Association between the Instruments and Plasma Adiponectin

Table S3 shows the information about the 47 SNPs and their associations with APN levels in our participants. Five SNPs, rs12495941, rs2339298, rs17244777, rs12051272, and rs12922394 located on ADIPOQ, RYR3, LOC101928392, CDH13, and CDH13, respectively, showed highly significant associations with plasma APN (*p* = 0.003, 0.005, 0.027, <0.001, and 0.002, respectively). Most SNPs were weak instruments based on their F value. Only rs12051272, located on CDH13, was a strong instrument (*f* value = 23.9).

Furthermore, we combined multiple independent SNPs into GRS as instruments in the present study, including the 5 independent SNPs significantly associated with APN levels (GRS_APN_), the 47 independent SNPs significantly associated with APN levels in the Asian population as determined from previous reports (GRS_All_), and the 16 independent SNPs which comprised GRS_All_-SNPs limited with a rigorous threshold (*p* < 5.0 × 10^−8^ for GWAS) (GRS_Limited_). These three instruments were all strong instruments for APN level (F values = 38.53, 64.78 and 44.68 for GRS_APN_, GRS_All_, and GRS_Limited_, respectively). GRS was associated with increments of 1-standard deviation (SD) of APN for GRS_APN_ (β = 0.09 (95% CI: 0.06 to 0.12, *p* = 2.17 × 10^−10^)), GRS_All_ (β  =  0.04 (95% CI: 0.03 to 0.05, *p* = 4.53 × 10^−16^)), and GRS_Limited_ (β  = 0.07 (95% CI: 0.05 to 0.09, *P* = 4.24 × 10^−11^)) in the base model. The R2 for GRS in association analysis with APN was between 3.2% and 5.2% (3.2%, 5.2%, and 3.4% for GRS_APN_, GRS_All_, and GRS_Limited_, respectively) (Table S3).

### 3.3. MR Analysis for the Effect of Adiponectin on DR Status

We estimated the combined effect size across multiple SNPs by the IVW method. The IV-estimated effect size of APN levels on DR was calculated for the different sets of genetic instruments. For each 1-SD increase in genetically induced plasma APN, the OR of with DR was β = 0.20 (95% CI: −0.46 to 0.85, *p* = 0.553) for GRS_APN_, 0.61 (95% CI: 0.10–1.13, *p* = 0.020) for GRS_All_, and 0.57 (95% CI: −0.06 to 1.20, *p* = 0.078) for GRS_Limited_ (Table 2). If the rs12495941 SNP located on the ADIPOQ gene was used as the IV, the β was 0.10 (95% CI: −1.32 to 1.52, *p* = 0.892). These estimates reflect the effect of genetically influenced APN levels on DR, and are assumed to be free from confounding factors.

### 3.4. Association between the Instruments and Potential Risk Factors

Genetic variants may affect the outcome of interesting through pathways other than the risk factor of interest. Therefore, the sensitivity analyses, including MR-Egger regression and weighted-median approach, were performed. For outcomes of the weighted-median method, APN-related SNPs showed no significance (*p* values = 0.731, 0.677, and 0.736 for 5 SNPs related to APN level, all 47 SNPs, and 16 limited SNPs, respectively). Furthermore, the intercepts of MR-Egger were not significant (*p* = 0.663 and 0.363 for 5 SNPs related to APN level and all 47 SNPs, respectively), except for GRS_Limited_ (*p* = 0.005). In addition, the associations between different GRSs and the potential confounding factors, including gender, SBP/DBP, glucose, HbA1c, TG, HDL, LDL, and BMI were investigated; and no significant results were found; except the association between GRS_APN_ and HDL (*p* = 0.0397) (Table S5). These results indicate absence of a directional pleiotropy effect of APN-related genetic instruments on potential DR risk factors.

## 4. Discussion

To the best of our knowledge, this is the first study to investigate the causal relationship between APN and DR in an East Asian population using an MR method. MR has been used to investigate the possible causal relationship of an intermediate trait (such as APN levels) with disease (such as DR), taking advantage of the random allocation of alleles at conception [21]. Since alleles are randomly allocated during gametogenesis and genotype is a fixed exposure, MR studies are not as vulnerable to confounding and reverse causality. This substantially improves causal inference from observational data. We report that genetically determined APN level is not associated with DR, by using different APN-GRS as IVs. The result from using GRS_All_ showed that an increasing APN level could be a risk factor for developing DR; however, when we limited the evaluation to SNPs on the ADPIOQ gene, GRS_APN_, or GRS_Limited_, the causal role of APN on DR was not identified. Hence, the evidence for the causal association between APN and DR among our study population in Taiwan is limited, and needs to be confirmed by other studies with larger sample sizes.

Since, APN is involved in different pathways related to diabetes, it has been suggested as a potential biomarker to predict DR risk in T2D [4,5,7]. However, observational epidemiological studies provide inconsistent findings on the association between APN levels and risk of DR [16,17,30], probably due to the presence of residual confounding factors. A meta-analysis of 19 studies including 1545 cases and 1502 controls, found a significant negative association between APN concentrations and the severity of DR in Han Chinese T2D [17]. Another meta-analysis of 3 cross-sectional studies, including 324 DR cases and 983 T2D controls, found APN levels to be higher in those with microvascular complications [16]. Most of these studies were cross-sectional observational studies which carried the possibility of selection bias.

The MR study makes 3 assumptions of the IV analysis to derive a valid interpretation [31,32]. First, the genetic markers should be strongly associated with the APN level. We explored the strength of the SNPs-APN association using the *f*-test. In our study, each SNP had a very small effect on the APN level (all SNPs had *f* values <  10, except rs12051272). Therefore, we combined multiple variants into the GRS, which based on the effect size of each variant, increased its statistical power. Three different GRS instruments were calculated, namely; the combination of 5 SNPs significantly related to APN level in our population (GRS_APN_), 47 SNPs reported by previous studies (GRS_All_), and 16 SNPs reported by previous studies with a rigorous threshold (*p* < 5.0 × 10^−8^ for GWAS, GRS_Limited_). From the results of the F test, all of the three GRS instruments used were strong instruments (F values = 38.53, 64.78, and 44.68 for GRS_APN_, GRS_All_, and GRS_Limited_; respectively) for APN level. The second assumption is that the genetic markers are independent of exposure-outcome confounders. In our study, several selected SNPs (rs328 and rs2925979) were associated with traits other than APN. Some of these traits had widely known associations with DR risk factors such as hypertension, metabolic disease, and smoking. We limited SNP associated with only the APN level to construct the conservative GRS (GRS_APN_). The outcome was β = 0.61 for the expanded GRS_All_, and 0.20 for the conservative GRS_APN_. We also investigated the association between GRSs of APN and the potential confounding factors (HbA1c, BMI, blood lipid) by the IVW method, and none of them were significant. Also, the confounded MR results could have occurred through the introduction of subgroups of different genetic ancestries. Our study population was a group of individuals who live in Taiwan and were of Han ancestry [23]. Hence, confounding based on population stratification was limited in our study. A third assumption was that the genetic markers should affect DR only through their effect on the APN level. We included two sensitivity analyses; namely the MR-Egger regression and the weighted-median approach to investigate the horizontal pleiotropic effect of IV. We found no evidence of genetic variants that may affect the outcome through pathways other than through the risk factor of interest (APN).

The level of APN is influenced by various factors such as the degree of obesity, age, blood lipid, gender, smoking [33], glucose level, kidney function, the form of APN measured, genetic background, and medications. In general, medication use was poorly reported in studies and none adjusted the results for medication use. Drugs such as agonists, thiazolidinediones [34,35], and dipeptidyl peptidase-4 inhibitors [36], can change APN levels [37]. Moreover, the effect of the adjunct therapies between medicines treated for comorbidities (e.g., fibrates for hyper-triglyceridemia) and antidiabetic drugs among diabetic patients due to multiple co-morbidities on blood APN level is unclear. As such, there is difficulty in establishing a causal relationship between plasma APN and diabetic complications in observational studies. In previous MR studies, an increase of APN would cause increased metabolic profiling [38,39] and insulin sensitivity [40]. The causal role of APN levels in CHD pathogenesis; however, has not been identified [20,41,42], and no causal role of APN levels in DR was confirmed in the present study.

We have recognized limitations in our study. The limited evidence of a causal relationship between APN and DR could be due to the relatively small sample size used in the MR analysis. Based on our results from a total of 1251 samples with a 5.2% proportion of variance (R2xz) and OR of 1.84 when using GRS_All_ as IV, the power was 75% with a 5% significance level (two-sided) [43]. The power was lower when using other more conservative IVs (GRS_APN_ or GRS_Limited_). The small proportion of contribution of the GRS to the variation of APN risk can also partly explain the null association. The weighted GRS explained 3.2% to 5.2% of the total variation in the APN risk. The selected loci were derived from a relatively small number of GWASs conducted in the Asian population compared to the European population. We recommend inclusion of more APN-related variants in future MR analyses, especially in Asian populations. Also, the validity of DR status was a concern. In the present study, the DR status was self-reported and a proportion of cases were confirmed by ophthalmologists. Among those subjects recruited from hospital, 82.6% of cases (362/438) and 40.5% (147/363) of controls had double confirmed their DR status by ophthalmologist. However, no validity information was able to be obtained for subjects from TWB (detailed information shown in Table S1). Therefore, some subjects who self-reported without DR status may actually have retinopathy, thus diluting the strength of any statistical association. When we limited to those subjects with confirmed DR status by ophthalmologists (362 cases and 147 controls), the β values of IVW method increased (Table S6).

## 5. Conclusions

In conclusion, we investigated the causal relationship between APN and DR in an East Asian population. The MR analysis lent limited evidence for the causal role of APN in DR development among Taiwanese diabetic patients. The observed direction of null association might provide new clues for future studies. Studies with large sample sizes from different ethnic backgrounds are warranted to further confirm our findings.

## Figures and Tables

**Table 1:** Demographics of the study population.

	Non-DR (*n* = 813)	DR (*n* = 438)	*p* Value
Gender			0.231
Male	436 (56.9%)	234 (53.4%)	
Female	350 (43.1%)	204 (46.6%)	
Age (years)	59.43 (11.84)	61.39 (10.35)	0.002 *
SBP	130.67 (18.22)	141.99 (19.82)	<0.001 *
DBP	75.82 (11.77)	80.02 (12.90)	<0.001 *
BMI	26.54 (4.32)	25.70 (4.17)	0.001 *
Fasting glucose	134.15 (48.72)	149.21 (68.58)	0.002 *
HbA1c	7.27 (1.40)	8.07 (1.78)	<0.001 *
TG	166.65 (141.86)	159.42 (125.32)	0.585
HDL	47.36 (20.03)	43.88 (12.75)	0.162
LDL	112.68 (36.43)	89.41 (31.14)	<0.001 *
Adiponectin #	2.49 (0.90)	3.51 (0.82)	<0.001 *

Values are presented as N (%) or mean ± standard deviation (SD). Abbreviation: DR, diabetic retinopathy; BMI, body mass index; HbA1c: hemoglobin A1c; SBP/DBP: systolic/diastolic blood pressure; HDL: high density lipoprotein; LDL: low density lipoprotein; TG: triglyceride. *p* value for chi square test or two independent samples *t* test. * represents *p* values less than 0.05. # represents natural logarithm transformation per SD.

**Table 2:** The association of adiponectin on diabetic retinopathy risk using Mendelian randomization.

	IVW				MR-egger				Weight Median			
	β	95%CI	90%CI	P	Intercept	95%CI	90%CI	P	β	95%CI	90%CI	P
5 SNPs	0.20	−0.35–0.76	−0.26–0.67	0.473	0.06	−0.22–0.35	−0.18–0.30	0.663	0.12	−0.56–0.79	−0.45–0.69	0.731
GRS_APN_	0.20	−0.46–0.85	−0.35–0.75	0.553	-	-	-	-	-	-	-	-
47 SNPs	0.46	0.003–0.91	0.08–0.84	0.049 *	0.02	−0.02–0.06	−0.02–0.05	0.363	0.14	−0.52–0.79	−0.41–0.69	0.677
GRS_All_	0.61	0.10–1.13	0.18–1.05	0.020 *	-	-	-	-	-	-	-	-
16 SNPs	0.44	−0.20–1.08	−0.10–0.98	0.181	0.11	0.03–0.19	0.05–0.18	0.005 *	0.12	−0.56–0.79	−0.45–0.69	0.736
GRS_Limited_	0.57	−0.06–1.20	0.04–1.10	0.078	-	-	-	-	-	-	-	-

Abbreviation: IVW, inverse-variance weighted; MR, Mendelian randomization; SNP, single nucleotide polymorphism; CI, confidence interval; GRS_APN_: 5 SNPs significantly associated with APN levels; GRS_All_: 47 SNPs significantly associated with APN levels in the Asian population from previous reports; GRS_Limited_: 16 independent SNPs which comprised GRS_All_-SNPs limited with a rigorous threshold (*p* < 5.0 × 10^−8^ for GWAS and * *p* < 0.05 for case-control or meta-analysis studies).

## Data Availability

The data presented in this study are available on request from the corresponding author. The data are not publicly available due to ethnical.

## Supplementary Materials

The following are available online at https://www.mdpi.com/2073-4425/12/1/17/s1, Table S1: The number of subjects from different sources, and with diagnoses confirmed by ophthalmologists. Table S2: Sixty-eight SNPs associated with blood APN levels among subjects with an Asian ancestry, identified from previous reports. Table S3: The association between the 47 identified SNPs with blood APN level and diabetic retinopathy status among the Taiwanese population. Table S4: Selected SNPs for each instrument variable. Figure S1: Flowchart for identifying genetic variants. Table S5: Association between the instruments and potential risk factors. Table S6: The association of adiponectin on diabetic retinopathy risk using Mendelian randomization. (Limit subjects confirmed by doctor).
