# Supplementary material for: Causal Relationship between Adiponectin and Diabetic Retinopathy: A Mendelian Randomization Study in an Asian Population
Source: Genes (Basel). 2020 Dec 24;12(1):17. doi: 10.3390/genes12010017 (PMC7823606; doi:10.3390/genes12010017)
Supplement: Supplementary file 1 [file genes-12-00017-s001.pdf]

Table S1. The number of subjects from different sources, and with diagnoses confirmed by ophthalmologists.

	Data Source	
	China Medical University	Taiwan Biobank
Total numbers	801	450
Cases # (# confirmed by doctor)	438 (362)	0 (0)
Controls # (# confirmed by doctor)	363 (147)	450 (0)

#: number

Table S2. Sixty-eight SNPs associated with blood APN levels among the Asian ancestry, identified from previous reports.

NO.	SNP	Gene	Chr.	Position (GRCh37)	Alleles	Study	Population	Effect alleles	Beta for Effect allele	*P*
1	rs12072620		1	190528074	C/T	GWAS [1]	Korean		-0.074	9.00×10^{-7}
2	rs1501501		1	190528445	T/C	GWAS [1]	Korean		-0.074	9.00×10^{-7}
3	rs10753929	KLHL12	1	202923178	T/C	GWAS [2]	Taiwan	T	0.118	0.03
4	rs2889921		1	222339638	G/A	GWAS [1]	Korean		-0.087	1.94×10^{-5}
5	rs7558386	MIR5702	2	227562139	A/G	Meta [3]	East Asians	A	-0.06	1.40×10^{-3}
6	rs12714975	LOC105376981	3	19085374	C/G	Meta [3]	East Asians	C	0.21	8.90×10^{-5}
7	rs1438545	ALCAM	3	105052404	G/A	GWAS [1]	Korean		0.089	6.29×10^{-7}
8	rs266719	KNG1	3	186501648	T/C	Meta [3]	East Asians	T	0.13	7.00×10^{-7}
9	rs10937273	ADIPOQ	3	186549695	A/G	Meta [3]	East Asians	A	0.15	1.80×10^{-22}
10	rs1656930	ADIPOQ	3	186552857	A/G	GWAS [4]	Japan		-0.066	3.40×10^{-2}
11	rs266729	ADIPOQ	3	186559474	G/C	Case-Control [5]	HK		-0.067	0.0037
12	rs182052	ADIPOQ	3	186560782	A/G	Case-Control [5]	HK		-0.097	0.0001
13	rs12495941	ADIPOQ	3	186568180	T/G	Case-Control [5]	HK		0.103	0.0001
14	rs2241766	ADIPOQ	3	186570892	G/T	Case-Control [6]	China	GG+GT	-0.195	0.014
15	rs11722604	TDO2	4	156864751	A/C	GWAS [1]	Korean		0.077	2.31×10^{-5}
16	rs4311394	ARL15	5	53300662	G/A	GWAS [2]	Taiwan	G	0.034	0.027
17	rs17156226		5	103505728	A/G	GWAS [1]	Korean		0.068	1.19×10^{-5}
18	rs12211360	HIVEP2	6	143119832	G/A	Meta [3]	East Asians	A	-0.21	5.50×10^{-6}
19	rs328	LPL	8	19819724	G/C	Case-Control [7]	Japan			0.034
20	rs10957333	LOC105375880	8	65941449	G/A	GWAS [1]	Korean		-0.08	1.63×10^{-5}
21	rs436753		8	88530861	T/C	GWAS [1]	Korean		0.063	6.30×10^{-6}
22	rs2817677	SLIT1	10	98821828	G/A	GWAS [1]	Korean		-0.058	2.34×10^{-5}
23	rs3943077	LOC105378522	10	122945086	G/A	Meta [3]	East Asians	A	0.09	3.00×10^{-14}

24	rs4936310	LOC105369489	11	110900760	A/T	GWAS [1]	Korean		0.066	4.30×10^{-6}
25	rs11168618	C12orf54	12	48933233	T/C	Meta [3]	East Asians	T	-0.1	1.20×10^{-7}
26	rs17251474	BTG1	12	92565318	G/C	GWAS [1]	Korean		-0.064	1.14×10^{-5}
27	rs10847980	HIP1R	12	123387922	G/T	Meta [3]	East Asians	T	-0.08	7.40×10^{-6}
28	rs1187415	CCDC92	12	124491529	G/C	Meta [3]	East Asians	C	-0.14	2.30×10^{-7}
29	rs4943398		13	37185064	A/T	GWAS [1]	Korean		-0.064	2.90×10^{-6}
30	rs7148411	OR4U1P	14	20559282	G/A	GWAS [1]	Korean		0.063	9.90×10^{-6}
31	rs2676077	RYR3	15	33605301	A/G	GWAS [8]	China			0.001
32	rs8035021	RYR3	15	33628840	T/C	GWAS [8]	China			0.001
33	rs2596181	RYR3	15	33651165	G/T	GWAS [8]	China			0.001
34	rs1975242	RYR3	15	33801207	A/G	GWAS [8]	China			0.001
35	rs2304380	RYR3	15	33855138	C/T	GWAS [8]	China			0.001
36	rs682639	RYR3	15	33866278	TC	GWAS [8]	China			0.001
37	rs2229116	RYR3	15	33905410	G/A	GWAS [8]	China			0.001
38	rs1495280	RYR3	15	33927764	C/T	GWAS [8]	China			0.001
39	rs8031151	RYR3	15	33938396	A/T	GWAS [8]	China			0.001
40	rs4780144	RYR3	15	33954652	C/T	GWAS [8]	China			0.001
41	rs716368	RYR3	15	33971926	C/T	GWAS [8]	China			0.001
42	rs2339298	RYR3	15	33987505	C/T	GWAS [8]	China			0.001
43	rs2293028	RYR3	15	34014913	C/T	GWAS [8]	China			0.001
44	rs8029059	RYR3	15	34030192	T/C	GWAS [8]	China			0.001
45	rs12901404	RYR3	15	34075300	T/C	GWAS [8]	China			0.001
46	rs2115747	RYR3	15	34094201	T/C	GWAS [8]	China			0.001
47	rs1036007	RYR3	15	34112906	C/A	GWAS [8]	China			0.001
48	rs2288614	RYR3	15	34113536	A/G	GWAS [8]	China			0.001
49	rs2278314	RYR3	15	34140735	A/G	GWAS [8]	China			0.001

50	rs7171526	TLN2	15	62709924	G/C	GWAS [1]	Korean		-0.078	1.19×10^{-5}
51	rs2925979	CMIP	16	81534790	T/C	Meta [3]	East Asians	T	-0.07	2.10×10^{-10}
52	rs17244777	LOC101928392	16	82602083	C/A	GWAS [1]	Korean			6.90×10^{-4}
53	rs11646213	CDH13	16	82642651	T/A	Case-Control [9]	Taiwan	AA		0.001
54	rs7200895	CDH13	16	82644606	T/C	GWAS [1]	Korean			2.05×10^{-8}
55	rs3852724	CDH13	16	82646094	C/A	GWAS [1]	Korean		0.057	6.23×10^{-7}
56	rs12596316	CDH13	16	82646152	G/A	GWAS [1]	Korean		-0.088	4.76×10^{-13}
57	rs3865185	CDH13	16	82646462	A/T	GWAS [1]	Korean		0.057	6.23×10^{-7}
58	rs3865186	CDH13	16	82646972	A/G	GWAS [1]	Korean		0.057	5.84×10^{-7}
59	rs3865188	CDH13	16	82650717	T/A	GWAS [1]	Korean		-0.309	1.67×10^{-15}
60	rs7193788	CDH13	16	82656160	G/A	GWAS [2]	Taiwan	G	0.254	4.88×10^{-5}
61	rs7204454	CDH13	16	82659194	C/G	GWAS [1]	Korean			1.78×10^{-16}
62	rs12444338	CDH13	16	82660155	T/G	Case-Control [9]	Taiwan	GG		2.23×10^{-11}
63	rs4783244	CDH13	16	82662268	T/G	GWAS [2]	Taiwan	G	0.346	7.57×10^{-9}
64	rs12051272	CDH13	16	82663288	T/G	Case-Control [9]	Taiwan	GG		1.57×10^{-10}
65	rs8047711	CDH13	16	82667671	A/G	GWAS [2]	Taiwan	G	0.323	4.10×10^{-6}
66	rs12922394	CDH13	16	82672327	T/C	GWAS [1]	Korean			1.19×10^{-8}
67	rs889140	AKR1B1P7	19	33889000	A/G	Meta[3]	East Asians	A	0.07	3.60×10^{-12}
68	rs6518702	SEC14L4	22	30948752	T/C	Meta [3]	East Asians	T	-0.08	5.30×10^{-4}

Abbreviations: No., number; SNP, single nucleotide polymorphism; APN, adiponectin; GWAS, genome-wide association study; Meta, meta-analysis; Chr., chromosome.

1. Jee, S.H.; Sull, J.W.; Lee, J.E.; Shin, C.; Park, J.; Kimm, H.; Cho, E.Y.; Shin, E.S.; Yun, J.E.; Park, J.W., et al. Adiponectin concentrations: a genome-wide association study. *American journal of human genetics* **2010**, *87*, 545-552, doi:10.1016/j.ajhg.2010.09.004.
2. Chung, C.M.; Lin, T.H.; Chen, J.W.; Leu, H.B.; Yang, H.C.; Ho, H.Y.; Ting, C.T.; Sheu, S.H.; Tsai, W.C.; Chen, J.H., et al. A genome-wide association study reveals a quantitative trait locus of adiponectin on CDH13 that predicts cardiometabolic outcomes. *Diabetes* **2011**, *60*, 2417-2423, doi:10.2337/db10-1321.

3. Wu, Y.; Gao, H.; Li, H.; Tabara, Y.; Nakatochi, M.; Chiu, Y.F.; Park, E.J.; Wen, W.; Adair, L.S.; Borja, J.B., et al. A meta-analysis of genome-wide association studies for adiponectin levels in East Asians identifies a novel locus near WDR11-FGFR2. *Human molecular genetics* **2014**, *23*, 1108-1119, doi:10.1093/hmg/ddt488.
4. Tanimura, D.; Shibata, R.; Izawa, H.; Hirashiki, A.; Asano, H.; Murase, Y.; Miyata, S.; Nakatochi, M.; Ouchi, N.; Ichihara, S., et al. Relation of a common variant of the adiponectin gene to serum adiponectin concentration and metabolic traits in an aged Japanese population. *European journal of human genetics : EJHG* **2011**, *19*, 262-269, doi:10.1038/ejhg.2010.201.
5. Ong, K.L.; Li, M.; Tso, A.W.; Xu, A.; Cherny, S.S.; Sham, P.C.; Tse, H.F.; Lam, T.H.; Cheung, B.M.; Lam, K.S. Association of genetic variants in the adiponectin gene with adiponectin level and hypertension in Hong Kong Chinese. *European journal of endocrinology* **2010**, *163*, 251-257, doi:10.1530/eje-10-0251.
6. Han, Y.; Zheng, Y.L.; Fan, Y.P.; Liu, M.H.; Lu, X.Y.; Tao, Q. Association of adiponectin gene polymorphism 45TG with gestational diabetes mellitus diagnosed on the new IADPSG criteria, plasma adiponectin levels and adverse pregnancy outcomes. *Clinical and experimental medicine* **2015**, *15*, 47-53, doi:10.1007/s10238-014-0275-8.
7. Fujiwara, S.; Kotani, K.; Sano, Y.; Matsuoka, Y.; Tsuzaki, K.; Domichi, M.; Kajii, E.; Sakane, N. S447X polymorphism in the lipoprotein lipase gene and the adiponectin level in the general population: results from the Mima study. *Journal of atherosclerosis and thrombosis* **2009**, *16*, 188-193, doi:10.5551/jat.e593.
8. Chang, Y.C.; Chiu, Y.F.; He, C.T.; Sheu, W.H.; Lin, M.W.; Seto, T.B.; Assimes, T.; Jou, Y.S.; Su, L.; Lee, W.J., et al. Genome-wide linkage analysis and regional fine mapping identified variants in the RYR3 gene as a novel quantitative trait locus for circulating adiponectin in Chinese population. *Medicine* **2016**, *95*, e5174, doi:10.1097/md.0000000000005174.
9. Teng, M.S.; Hsu, L.A.; Wu, S.; Sun, Y.C.; Juan, S.H.; Ko, Y.L. Association of CDH13 genotypes/haplotypes with circulating adiponectin levels, metabolic syndrome, and related metabolic phenotypes: the role of the suppression effect. *PloS one* **2015**, *10*, e0122664, doi:10.1371/journal.pone.0122664.

Table S3. The association between the 47 identified SNPs with blood APN level and diabetic retinopathy status among Taiwanese population.

	rs ID	Gene	Chr.	Position (GRCh37)	Alleles*	MAF	HWE	Association with outcome		Association with adiponectin		
								OR (95%CI)	*P*	β (95%CI)	*P*	*F* statistic
1	rs12072620		1	190528074	T/C	0.1531	0.8005	1.14 (0.90,1.44)	0.2772	0.01 (-0.10,0.12)	0.8069	0.06
2	rs10753929	KLHL12	1	202923178	T/C	0.0972	0.7868	1.09 (0.83,1.43)	0.5296	0.09 (-0.04,0.22)	0.1716	1.93
3	rs2889921		1	222339638	G/A	0.2116	0.3561	1.04 (0.85,1.27)	0.7056	0.003 (-0.09,0.10)	0.9497	0.00
4	rs7558386	MIR5702	2	227562139	A/G	0.3278	0.5622	0.96 (0.81,1.15)	0.6802	0.003 (-0.08,0.09)	0.9370	0.01
5	rs12714975	LOC105376981	3	19085374	C/G	0.0215	0.2993	1.11 (0.65,1.89)	0.7002	0.17 (-0.09,0.43)	0.1888	1.80
6	rs1438545	ALCAM	3	105052404	G/A	0.0898	0.8118	0.89 (0.67,1.20)	0.4602	0.05 (-0.09,0.19)	0.4714	0.53
7	rs266719	KNG1	3	186501648	T/C	0.0996	0.4183	1.16 (0.88,1.52)	0.2869	0.04 (-0.10,0.17)	0.5974	0.30
8	rs12495941	ADIPOQ	3	186568180	T/G	0.4139	0.8535	1.01 (0.85,1.20)	0.8919	0.12 (0.04,0.20)	0.0038	7.95
9	rs11722604	TDO2	4	156864751	C/A	0.1027	0.3563	1.09 (0.83,1.43)	0.5331	0.03 (-0.10,0.16)	0.6622	0.20
10	rs4311394	ARL15	5	53300662	A/G	0.449	0.0493	0.88 (0.75,1.04)	0.1450	0.03 (-0.05,0.10)	0.5246	0.39
11	rs17156226		5	103505728	G/A	0.1796	0.4435	1.30 (1.03,1.63)	0.0261	0.05 (-0.06,0.16)	0.3552	1.00
12	rs12211360	HIVEP2	6	143119832	A/G	0.0246	0.4721	0.83 (0.50,1.36)	0.4511	0.13 (-0.11,0.37)	0.2981	1.12
13	rs328	LPL	8	19819724	G/C	0.0935	0.3838	0.95 (0.71,1.25)	0.6983	-0.02 (-0.15,0.12)	0.8172	0.05
14	rs10957333	LOC105375880	8	65941449	G/A	0.1248	0.2854	0.83 (0.64,1.08)	0.1689	0.02 (-0.10,0.14)	0.7745	0.08
15	rs436753		8	88530861	T/C	0.3118	0.0091	0.98 (0.83,1.17)	0.8579	0.06 (-0.02,0.14)	0.1662	1.85
16	rs2817677	SLIT1	10	98821828	A/G	0.1716	0.316	1.12 (0.90,1.38)	0.3037	-0.01 (-0.11,0.09)	0.8748	0.03
17	rs3943077	LOC105378522	10	122945086	A/G	0.4096	0.3381	1.15 (0.97,1.36)	0.0973	0.04 (-0.04,0.12)	0.3353	0.95
18	rs4936310	LOC105369489	11	110900760	T/A	0.2294	0.5233	1.13 (0.92,1.37)	0.2422	0.03 (-0.07,0.12)	0.5529	0.32
19	rs11168618	C12orf54	12	48933233	C/T	0.1451	0.044	1.42 (1.09,1.84)	0.0083	0.06 (-0.05,0.18)	0.2914	1.05
20	rs17251474	BTG1	12	92565318	G/C	0.2362	0.2714	0.97 (0.80,1.18)	0.7555	0.06 (-0.03,0.15)	0.2219	1.58
21	rs10847980	HIP1R	12	123387922	G/T	0.1716	0.0466	1.12 (0.91,1.39)	0.2723	0.03 (-0.07,0.13)	0.5239	0.38
22	rs1187415	CCDC92	12	124491529	G/C	0.0695	0.1125	1.06 (0.77,1.46)	0.7367	0.11 (-0.04,0.27)	0.1639	1.71

23	rs4943398		13	37185064	T/A	0.203	0.4496	1.03 (0.84,1.26)	0.7972	0.01 (-0.08,0.11)	0.7673	0.09
24	rs7148411	OR4U1P	14	20559282	G/A	0.3621	0.6982	1.05 (0.89,1.25)	0.5462	0.07 (-0.01,0.15)	0.1062	2.74
25	rs2676077	RYR3	15	33605301	A/G	0.3702	0.7136	1.08 (0.91,1.28)	0.3756	0.04 (-0.05,0.12)	0.3829	0.75
26	rs2596181	RYR3	15	33651165	G/T	0.4514	0.7413	1.002 (0.85,1.18)	0.9797	0.02 (-0.06,0.10)	0.6385	0.23
27	rs1975242	RYR3	15	33801207	A/G	0.3106	0.5753	0.95 (0.80,1.14)	0.5925	0.04 (-0.04,0.13)	0.3262	0.90
28	rs2304380	RYR3	15	33855138	C/T	0.0855	0.3838	1.05 (0.77,1.41)	0.7740	-0.02 (-0.17,0.12)	0.7439	0.11
29	rs2229116	RYR3	15	33905410	G/A	0.1544	0.8662	0.96 (0.77,1.21)	0.7489	0.05 (-0.06,0.16)	0.4080	0.73
30	rs1495280	RYR3	15	33927764	T/C	0.3561	0.3658	1.07 (0.90,1.27)	0.4240	-0.004 (-0.09,0.08)	0.9299	0.01
31	rs8031151	RYR3	15	33938396	A/T	0.3165	0.7993	0.88 (0.73,1.06)	0.1680	0.02 (-0.07,0.10)	0.7244	0.13
32	rs716368	RYR3	15	33971926	T/C	0.3272	0.5208	0.96 (0.80,1.14)	0.6198	0.03 (-0.05,0.12)	0.4263	0.65
33	rs2339298	RYR3	15	33987505	T/C	0.3346	0.6366	1.07 (0.90,1.27)	0.4545	0.12 (0.03,0.20)	0.0056	7.54
34	rs2293028	RYR3	15	34014913	C/T	0.4403	0.3444	0.98 (0.82,1.15)	0.7698	-0.01 (-0.09,0.07)	0.8983	0.02
35	rs8029059	RYR3	15	34030192	C/T	0.3524	0.7542	1.09 (0.92,1.30)	0.3140	0.02 (-0.06,0.10)	0.6314	0.25
36	rs12901404	RYR3	15	34075300	C/T	0.3831	0.8411	1.14 (0.96,1.35)	0.1353	0.04 (-0.04,0.12)	0.3181	1.02
37	rs2115747	RYR3	15	34094201	T/C	0.4034	0.4961	1.06 (0.90,1.25)	0.4909	0.002 (-0.08,0.08)	0.9538	0.00
38	rs1036007	RYR3	15	34112906	C/A	0.2417	0.9248	0.95 (0.79,1.15)	0.6259	0.01 (-0.08,0.10)	0.7701	0.09
39	rs2278314	RYR3	15	34140735	A/G	0.3161	0.0564	0.97 (0.82,1.15)	0.7358	0.02 (-0.06,0.10)	0.5954	0.28
40	rs7171526	TLN2	15	62709924	G/C	0.1218	0.3163	0.85 (0.66,1.11)	0.2423	0.003 (-0.12,0.13)	0.9657	0.00
41	rs2925979	CMIP	16	81534790	T/C	0.4342	0.6968	1.06 (0.90,1.25)	0.4917	0.01 (-0.06,0.09)	0.7176	0.12
42	rs17244777	LOC101928392	16	82602083	A/C	0.2528	0.2608	1.06 (0.88,1.28)	0.5574	0.10 (0.01,0.19)	0.0277	4.56
43	rs7204454	CDH13	16	82659194	G/C	0.3565	0.179	0.98 (0.83,1.16)	0.8538	-0.08 (-0.16,0.004)	0.0634	3.58
44	rs12051272	CDH13	16	82663288	G/T	0.3093	0.844	1.03 (0.86,1.22)	0.7712	0.21 (0.13,0.30)	4.49×10^{-7}	23.90
45	rs12922394	CDH13	16	82672327	C/T	0.2232	0.4766	1.004 (0.82,1.22)	0.9704	0.14 (0.05,0.24)	0.0029	8.52
46	rs889140	AKR1B1P7	19	33889000	G/A	0.4416	0.6212	1.24 (1.05,1.47)	0.0097	0.04 (-0.04,0.12)	0.3074	1.09
47	rs6518702	SEC14L4	22	30948752	C/T	0.2503	0.2767	1.15 (0.94,1.39)	0.1660	0.02 (-0.07,0.11)	0.7176	0.14
	**GRS_{APN}**							1.02 (0.96,1.08)	0.5528	0.09 (0.06,0.12)	2.17×10^{-10}	38.53

**GRS_{All}**	1.03 (1.00,1.05)	0.0202	0.04 (0.03,0.05)	4.53×10^{-16}	64.78
**$GRS_{Limited}$**	1.04 (1.00,1.08)	0.078	0.07 (0.05,0.09)	4.24×10^{-11}	44.68

Abbreviations: SNP, single nucleotide polymorphism; APN, adiponectin; Chr., chromosome; MAF, minor allele frequency; HWE, Hardy-Weinberg equilibrium; OR, odds ratio; CI, confidence interval; GRS, genetic risk score. *The first allele is the effect allele for adiponectin.

GRS_{APN}: 5 SNPs significantly associated with APN levels.

GRS_{All}: 47 SNPs significantly associated with APN levels in Asian population from previous reports

$GRS_{Limited}$: 16 independent SNPs which comprised GRS_{All}-SNPs limited with a rigorous threshold ($P < 5.0 \times 10^{-8}$ for GWAS and $P < 0.05$ for case-control or meta-analysis studies)

Table S4. Selected SNPs for each instrument variable.

	rs ID	Gene	Study type	GRS$_{APN}$ (5 SNPs)	GRS$_{All}$ (47 SNPs)	GRS $_{Limited}$ (16 SNPs)
1	rs12072620		GWAS		✓	
2	rs10753929	KLHL12	GWAS		✓	
3	rs2889921		GWAS		✓	
4	rs7558386	MIR5702	Meta		✓	✓
5	rs12714975	LOC105376981	Meta		✓	✓
6	rs1438545	ALCAM	GWAS		✓	
7	rs266719	KNG1	Meta		✓	✓
8	rs12495941	ADIPOQ	Case-Control	✓	✓	✓
9	rs11722604	TDO2	GWAS		✓	
10	rs4311394	ARL15	GWAS		✓	
11	rs17156226		GWAS		✓	
12	rs12211360	HIVEP2	Meta		✓	✓
13	rs328	LPL	Case-Control		✓	✓
14	rs10957333	LOC105375880	GWAS		✓	
15	rs436753		GWAS		✓	
16	rs2817677	SLIT1	GWAS		✓	
17	rs3943077	LOC105378522	Meta		✓	✓
18	rs4936310	LOC105369489	GWAS		✓	
19	rs11168618	C12orf54	Meta		✓	✓
20	rs17251474	BTG1	GWAS		✓	
21	rs10847980	HIP1R	Meta		✓	✓
22	rs1187415	CCDC92	Meta		✓	✓
23	rs4943398		GWAS		✓	
24	rs7148411	OR4U1P	GWAS		✓	
25	rs2676077	RYR3	GWAS		✓	
26	rs2596181	RYR3	GWAS		✓	
27	rs1975242	RYR3	GWAS		✓	
28	rs2304380	RYR3	GWAS		✓	
29	rs2229116	RYR3	GWAS		✓	
30	rs1495280	RYR3	GWAS		✓	
31	rs8031151	RYR3	GWAS		✓	
32	rs716368	RYR3	GWAS		✓	
33	rs2339298	RYR3	GWAS	✓	✓	
34	rs2293028	RYR3	GWAS		✓	
35	rs8029059	RYR3	GWAS		✓	
36	rs12901404	RYR3	GWAS		✓	
37	rs2115747	RYR3	GWAS		✓	
38	rs1036007	RYR3	GWAS		✓	
39	rs2278314	RYR3	GWAS		✓	
40	rs7171526	TLN2	GWAS		✓	

41	rs2925979	CMIP	Meta		✓	✓
42	rs17244777	LOC101928392	GWAS	✓	✓	
43	rs7204454	CDH13	GWAS		✓	✓
44	rs12051272	CDH13	Case-Control	✓	✓	✓
45	rs12922394	CDH13	GWAS	✓	✓	✓
46	rs889140	AKR1B1P7	Meta		✓	✓
47	rs6518702	SEC14L4	Meta		✓	✓

Abbreviations: SNP, single nucleotide polymorphism; GRS, genetic risk score; GWAS, genome-wide association study; Meta, meta-analysis; APN, adiponectin.

GRS_{APN}: 5 SNPs significantly associated with APN levels

GRS_{All}: 47 SNPs significantly associated with APN levels in Asian population from previous reports

$GRS_{Limited}$: 16 independent SNPs which comprised GRS_{All}-SNPs limited with a rigorous threshold (P < 5.0 x 10^{-8} for GWAS and P < 0.05 for case-control or meta-analysis studies)

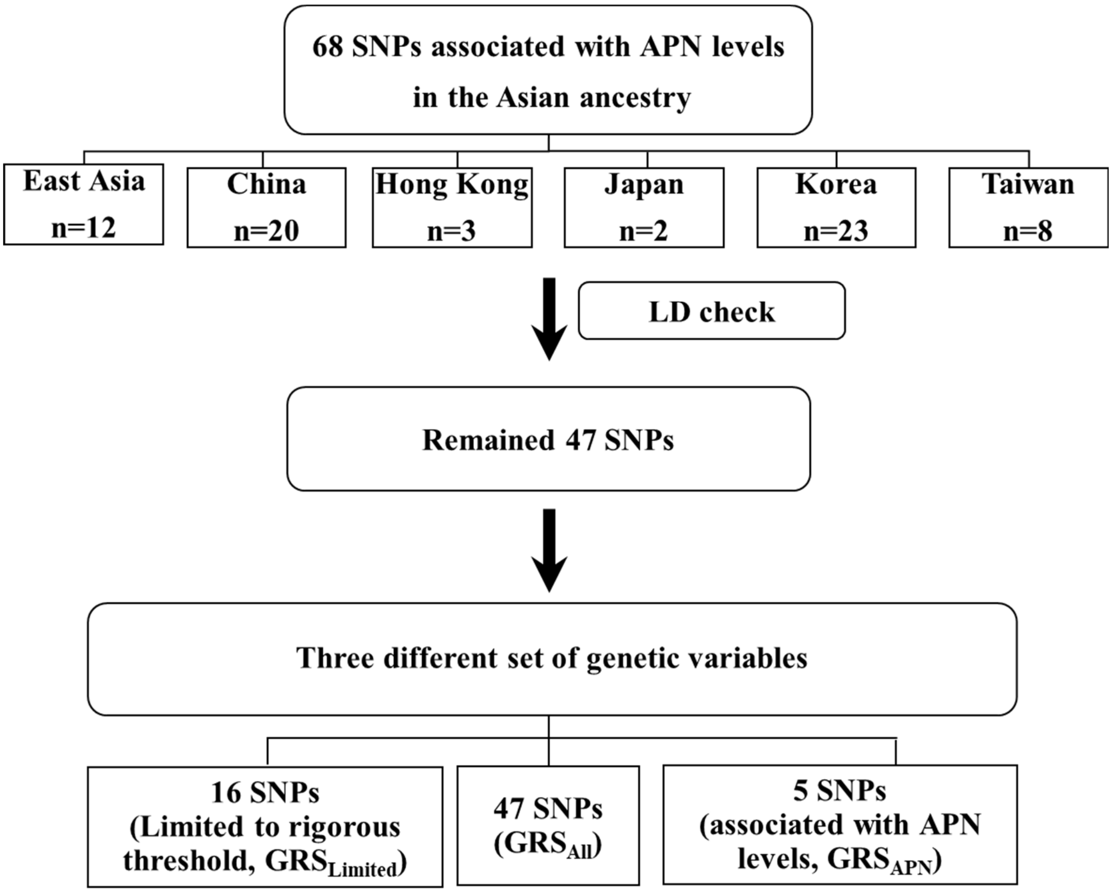

Figure S1: Flowchart for identifying genetic variants.

Table S5. Association between the instruments and potential risk factors.

Confounder	GRS_{APN}		GRS_{All}		$GRS_{Limited}$	
	Beta	*P*	Beta	*P*	Beta	*P*
gender	-0.03	0.3021	0.00	0.7076	-0.01	0.6357
SBP	-0.16	0.5628	0.04	0.6688	0.03	0.8623
DBP	-0.01	0.9723	-0.03	0.6293	0.01	0.9518
BMI	0.12	0.0548	0.03	0.2669	0.08	0.0610
glucose	-0.35	0.6761	-0.16	0.6078	-0.32	0.5930
HbA1c	0.03	0.1693	0.01	0.1740	0.01	0.4313
TG	-0.47	0.8453	-0.62	0.4772	-0.48	0.7779
HDLC	0.78	0.0397*	0.21	0.1319	0.32	0.2425
LDLC	-0.005	0.9943	-0.08	0.7262	-0.04	0.9300

Abbreviations: GRS, genetic risk score; APN, adiponectin; SBP, systolic blood pressure; DBP, diastolic blood pressure; BMI, body mass index; HbA1c: hemoglobin A1c; TG: triglyceride; HDL: high density lipoprotein; LDL: low density lipoprotein.

GRS_{APN}: 5 SNPs significantly associated with APN levels

GRS_{All}: 47 SNPs significantly associated with APN levels in Asian population from previous reports

$GRS_{Limited}$: 16 independent SNPs which comprised GRS_{All}-SNPs limited with a rigorous threshold ($P < 5.0 \times 10^{-8}$ for GWAS and $P < 0.05$ for case-control or meta-analysis studies)

Table S6. The association of adiponectin on diabetic retinopathy risk using Mendelian randomization. (Limit subjects confirmed by doctor)

	IVW				MR-egger				Weight Median			
	**Beta**	**95%CI**	**90%CI**	**P**	**Intercept**	**95%CI**	**90%CI**	**P**	**Beta**	**95%CI**	**90%CI**	**P**
5 SNPs	0.77	-0.02-1.56	0.11-1.43	0.056	0.05	-0.38-0.47	-0.31-0.40	0.834	0.60	-0.38-1.58	-0.23-1.43	0.232
GRS_{APN}	0.76	-0.17-1.69	--0.02-1.54	0.109	-	-	-	-	-	-	-	-
47 SNPs	0.91	0.41-1.42	0.49-1.34	< 0.001*	0.07	-0.001-0.15	0.01-0.14	0.053	0.70	-0.09-1.49	0.04-1.37	0.083
GRS_{GWAS}	1.15	0.54-1.76	0.64-1.66	< 0.001*	-	-	-	-	-	-	-	-
16 SNPs	0.94	0.28-1.60	0.38-1.49	0.006*	0.13	-0.01-0.26	0.01-0.24	0.068	0.70	-0.21-1.61	-0.06-1.46	0.131
$GRS_{Limited}$	0.95	0.20-1.71	0.32-1.58	0.013*	-	-	-	-	-	-	-	-

Abbreviation: IVW, inverse-variance weighted; MR, Mendelian randomization; SNP, single nucleotide polymorphism; CI, confidence interval; GRS_{APN}: 5 SNPs significantly associated with APN levels; GRS_{All}: 47 SNPs significantly associated with APN levels in Asian population from previous reports; $GRS_{Limited}$: 16 independent SNPs which comprised GRS_{All}-SNPs limited with a rigorous threshold (P < 5.0 x 10-8 for GWAS and P < 0.05 for case-control or meta-analysis studies).
